# Generalized herpes zoster and cutaneous metastasis during chemotherapy for non‐small cell lung cancer: A case report

**DOI:** 10.1111/1759-7714.13722

**Published:** 2020-10-28

**Authors:** Naoya Yasokawa, Yuri Yasuda, Houhi Chin, Koji Kurose, Yumi Aoyama, Toru Oga

**Affiliations:** ^1^ Department of Respiratory Medicine Kawasaki Medical School Kurashiki Japan; ^2^ Department of Dermatology Kawasaki Medical School Kurashiki Japan

**Keywords:** Cutaneous metastasis, generalized herpes zoster, non‐small cell lung cancer

## Abstract

Although herpes zoster is known to occur in some patients with lung cancer, generalized (disseminated) herpes zoster is an uncommon form whereby hematogenous dissemination of the virus occurs and leads to the development of widespread cutaneous lesions. Similarly, skin is an uncommon site of metastasis in patients with lung cancer. Here, we report a clinical case of a 53‐year‐old male patient who developed generalized herpes zoster during chemotherapy for non‐small cell lung cancer (squamous cell carcinoma) and subsequently developed cutaneous metastasis of lung cancer after generalized herpes zoster was cured by treatment with intravenous aciclovir. The coincidence of these two conditions, generalized herpes zoster and cutaneous metastasis, in the patient during lung cancer treatment might be associated with an impaired or dysregulated immune system partly due to repeated chemotherapy, indicating a poor prognosis. Close observation and accurate diagnosis of changes in the skin of patients with lung cancer are important when evaluating their immune status and considering their therapy and prognosis.

## Graphical abstract

A case of generalized herpes zoster and cutaneous metastasis found in a lung cancer patient undergoing chemotherapy

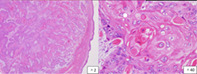

## Introduction

Herpes zoster, which is caused by reactivation of the varicella‐zoster virus (VZV), occurs in immunocompromised patients such as cancer patients and is related to their disease or treatments.^1^, ^2^ Herpes zoster has been previously reported in some patients with lung cancer.^1^, ^2^, ^3^ Reactivated virus spreads along the sensory nerve to the dermatome; however, generalized (disseminated) herpes zoster, in which the virus disseminates hematogenously to widespread cutaneous lesions, occurs in only about 2%–5% of herpes zoster cases.^4^, ^5^

The skin is an uncommon site of metastasis from internal malignancies. The overall incidence of cutaneous involvement is approximately 5% and may indicate advanced disease and a poor prognosis.^6^ Cutaneous metastasis of lung cancer is also rare.^7^, ^8^ Here, we report a patient who developed generalized herpes zoster during chemotherapy for non‐small cell lung cancer (NSCLC) and who subsequently developed cutaneous metastasis of lung cancer after generalized herpes zoster was cured. Herpes zoster is associated with cancer risk.^9^, ^10^, ^11^ The occurrence of two rare conditions, generalized herpes zoster and cutaneous metastasis, in the same patient should not be considered a chance finding, as it might be indicative of immunosuppression.

## Case report

A previously healthy 53‐year‐old man was admitted to our respiratory department with a history of exertional dyspnea and left shoulder pain for eight weeks. He had no underlying disease, no surgical history and no regular medications, but had smoked two packs a day between the ages of 14–40 years. Chest computed tomography (CT) revealed a 36 mm mass in the left S^3^ area. Bronchoscopy was performed, and he was diagnosed with non‐small cell lung cancer (squamous cell carcinoma) (cT2aN2M0, cStage IIIA). After first‐line chemotherapy with weekly carboplatin and paclitaxel plus radiation therapy (60 Gy), 14 cycles of second‐line chemotherapy with durvalumab were performed. However, because the lung cancer indicated progressive disease (PD), the third‐line chemotherapy was changed to docetaxel. F18‐fluorodeoxyglucose (FDG)‐positron emission tomography (PET)/CT indicated increased primary tumor, left pleural effusion and left subclavian lymphadenopathy. Biopsy of left subclavian lymphadenopathy was performed with a subsequent diagnosis of metastasis of squamous cell carcinoma, indicating a PD.

**Figure 1:**
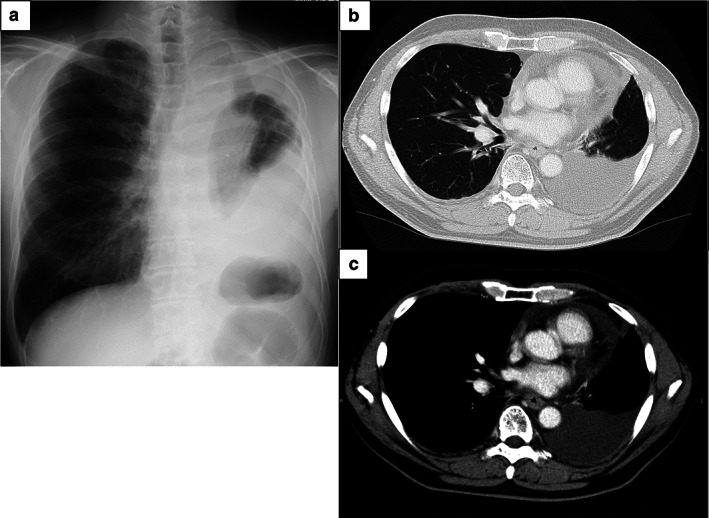
(**a**) Chest X‐ray showed decreased permeability in the lower left lung field and unaffected trachea; and (**b**–**c**) chest computed tomography (CT) scan showed a massive left pleural effusion and pericardial effusion.

The patient was hospitalized for the fourth‐line chemotherapy. During the first to fourth‐line chemotherapy, he was hospitalized for 7 to 14 days and then discharged for 7 to 14 days, for each chemotherapy course. The total length of his hospital stay before the fourth‐line chemotherapy was 203 days. During that period, the only adverse event was grade 1 radiation pulmonary inflammation (CTCAE 4.0) after the first‐line chemotherapy plus radiation therapy. Chest X‐ray showed extensive opacification in the left lung with massive pleural effusion indicated by chest CT (Fig 1a–c). Results of the blood test at this time were as follows; white blood cells 8150/μL; hemoglobin 13.2 g/dL; lactate dehydrogenase (LDH) 197 g/dL; total protein (TP) 6.9 g/dL; albumin 3.6 g/dL; globulin 3.2 g/dL; cholinesterase 297 U/L; and creatinine 0.75 mg/dL. Pale yellow exudative pleural effusion was observed (LDH 129 g/dL; TP 4.9 g/dL; albumin 2.7 g/dL; glucose 96 mg/dL; and lymphocytes 74.4%).

**Figure 2:**
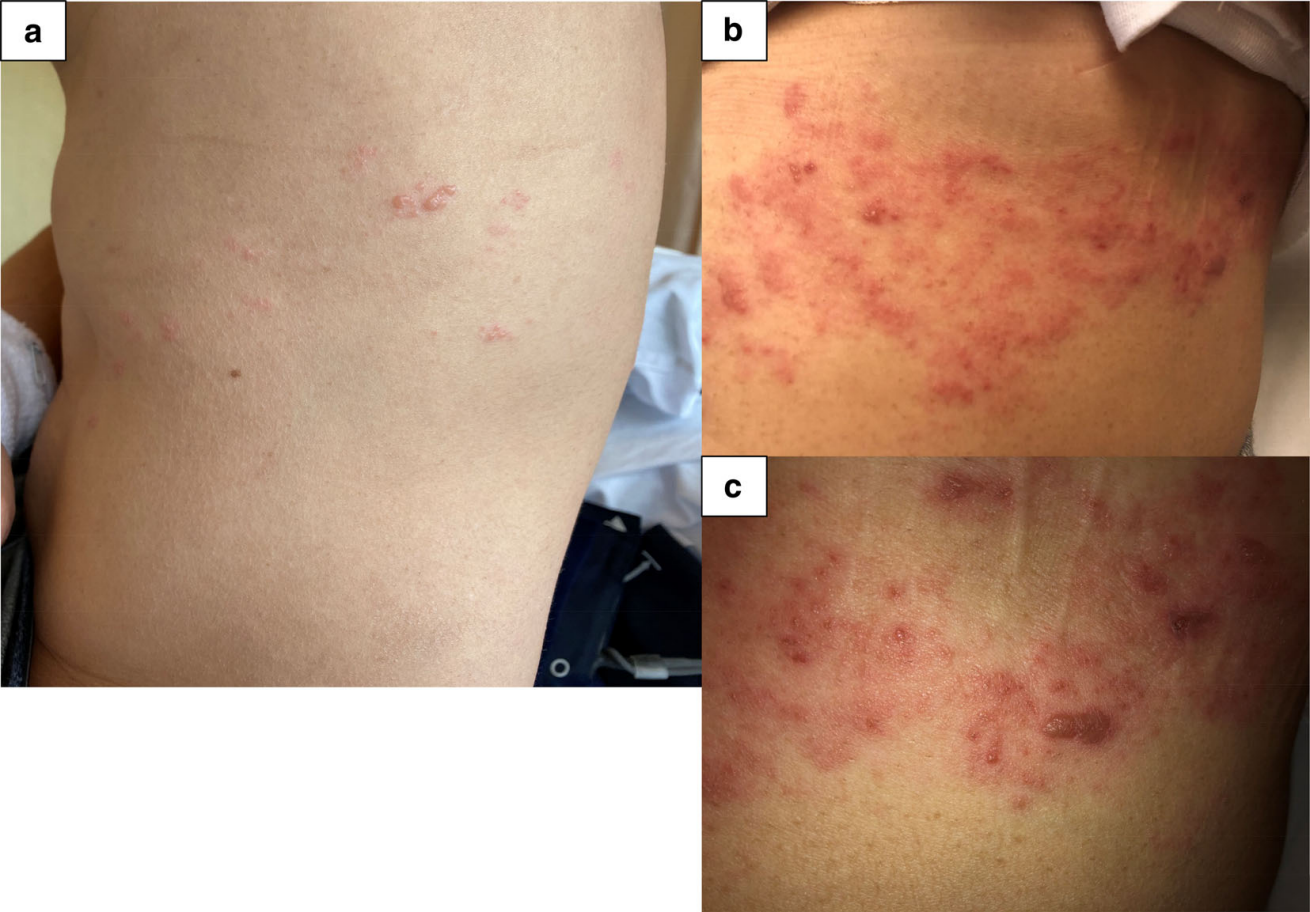
Exanthema with vesicles present in the left lateral region of the chest of the patient. (a) At diagnosis and (b) and (c) three days later.

On the night of the day of hospitalization, exanthema with vesicles was evident on the left lateral region of his chest (Fig 2a). Because herpes zoster was suspected, we administered valaciclovir hydrochloride 3000 mg orally daily. However, three days later, the exanthema with vesicles worsened (Fig 2b, c), and also appeared on his right wrist. Because the Tzanck smear test for the exanthema was positive, he was diagnosed as having generalized herpes zoster. Valaciclovir hydrochloride was stopped and aciclovir 750 mg intravenously daily was administered. The exanthema improved and we withdrew aciclovir eight days later. Then, he was discharged.

**Figure 3:**
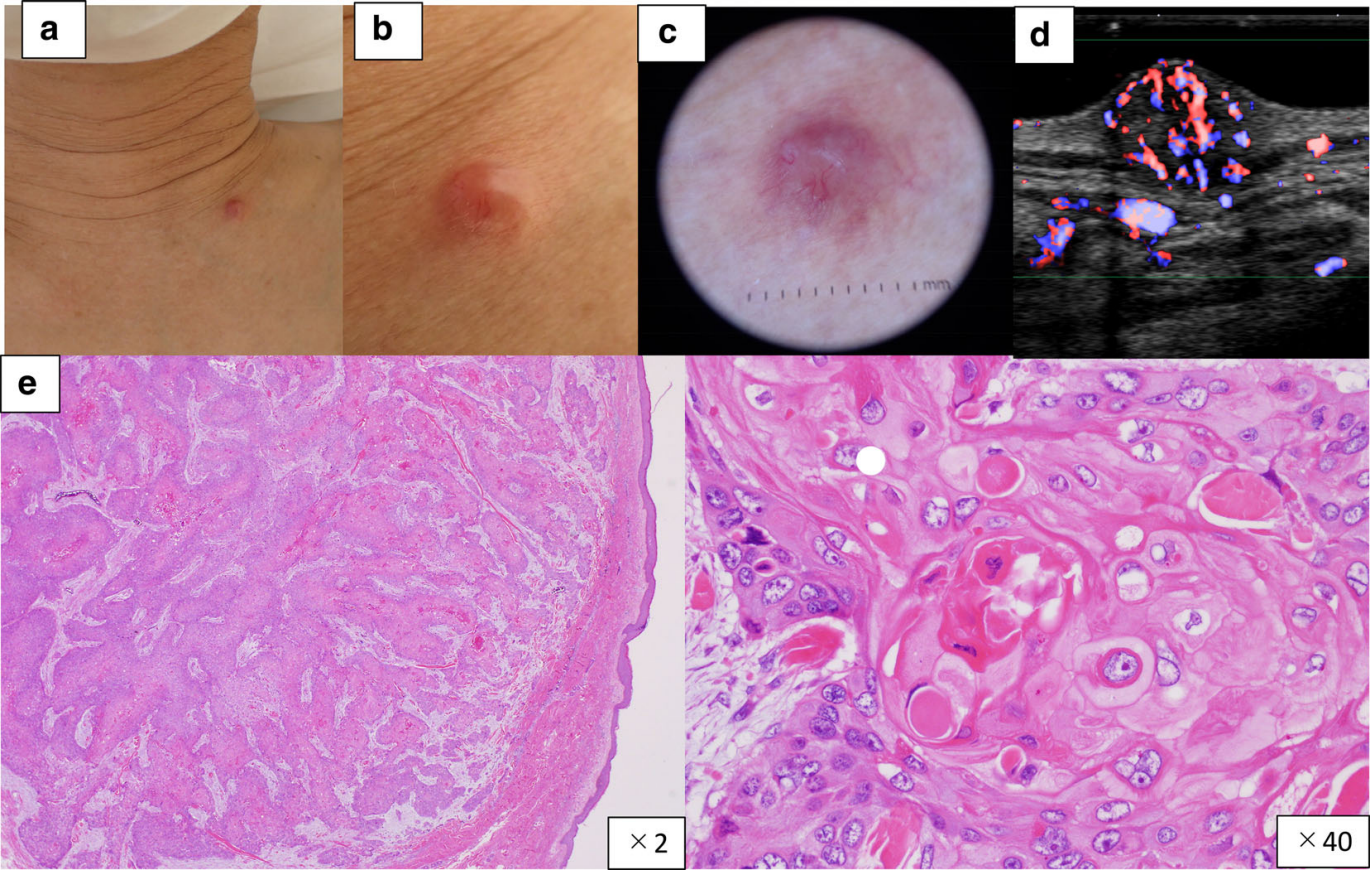
(**a**–**c**) A 3 mm subcutaneous nodule was present in the left sternal clavicle (bone); and (**d**) ultrasonography revealed cutaneous metastasis of lung cancer. (**e**) Histopathology indicated the nodule was formed mainly in the dermis to the subcutaneous tissue, with atypical cells forming solid alveolar nests (H&E). In addition, there was some continuity with the epidermis, with cancer pearls present in the alveolar nest.

However, two weeks later, he was rehospitalized suffering from the effects of the chemotherapy. A 3 mm subcutaneous nodule was observed in the left sternal clavicle (bone) (Fig 3a–c). Ultrasonography indicated a hypoechoic mass in the dermis and subcutaneous tissue. The boundaries were unclear, the contours were irregular, and blood flow signals were abundant (Fig 3d). A biopsy indicated a diagnosis of cutaneous metastasis of squamous cell carcinoma (Fig 3e). This was surgically removed because the patient felt pain there.

**Figure 4:**
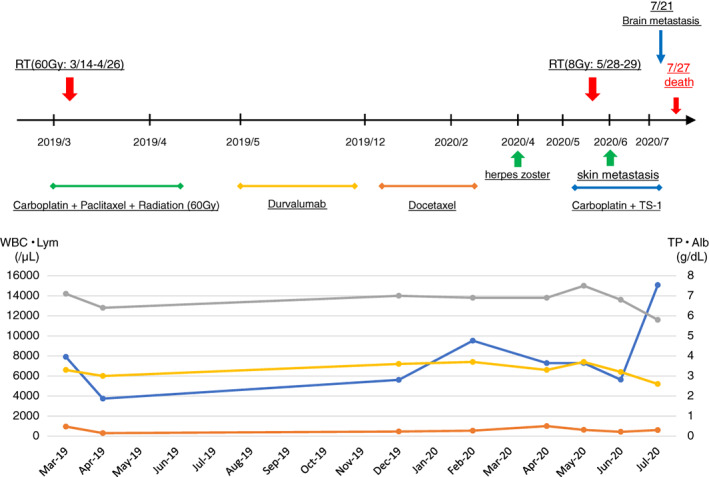
The clinical course of the patient (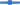) WBC (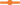) Lym (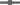) TP (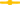) Alb.

About three weeks later, during the fourth‐line chemotherapy, he died of respiratory failure due to progressive lung cancer and massive pleural effusion. The clinical course of this patient is summarized in Fig 4.

## Discussion

Associations between the incidence of herpes zoster and malignancies have been reported.^1^, ^2^ Hata *et al*.^2^ reported that among 1410 patients with lung cancer, 35 (2.5%) developed herpes zoster. The incidence of herpes zoster in solid tumors is lower than hematological cancer.^1^, ^12^ Moreover, generalized herpes zoster, where VZV disseminates hematogenously from dorsal root ganglia cells to distant parts of the body, is uncommon.^4^ Its risk is increased in immunosuppressed patients. Our patient received repeated chemotherapy plus radiation therapy and chest drainage to treat pleural effusion. Physical trauma is a common cause of herpes zoster,^13^ indicating chest drainage or thoracentesis might have affected the incidence.

As with other internal malignancies, cutaneous metastasis from lung cancer is rare; for example, 1.7% of 1223 cases have been reported in the USA,^14^ 1.78% of 1292 cases in Taiwan,^7^ and 2.8% of 579 cases in Japan.^8^ The most common malignancies that metastasize to the skin are lung cancer in men, and breast cancer in women.^6^ The estimated mean survival after a diagnosis of cutaneous metastases has been reported to be 50% at six months,^6^ and the median survival of 16 Japanese cases of skin metastasis from lung cancer approximately four months,^8^ which is compatible with the present case. Cutaneous metastasis is typically located on the thorax, abdomen, head/neck, and scalp.^6^, ^15^ Some studies have reported that adenocarcinoma was the highest among cutaneous metastases from different histological types of lung cancer.^7^, ^15^ Clinical suspicion of cutaneous metastasis is highly important.

These two rare conditions of generalized herpes zoster and subsequent cutaneous metastasis might be associated with impaired or dysregulated immunity of the host. Cellular immune function is critical for suppressing VZV replication and carcinogenesis.^16^ When the cellular immune function is impaired, it causes an eruption of herpes zoster, which may be generalized. Under these circumstances, tumor immunity also deteriorates, promoting cancer, for example cutaneous metastasis in the present case. Repeated chemotherapy in this patient might have contributed to these conditions. Longitudinal epidemiological studies indicated herpes zoster has been found to be associated with increased risk of some types of cancer^9^, ^10^, ^11^ and that it might be an indicator of occult cancer. Hospitalization for herpes zoster has been reported to be associated with a risk of several types of cancer, indicating a poor prognosis. Thus, based on our experience and previous studies, we suggest the early detection of cancer metastasis or occult cancer is critical when a patient with lung cancer has generalized herpes zoster.

In conclusion, generalized herpes zoster and subsequent cutaneous metastasis during chemotherapy should not be regarded as a coincidence of two rare conditions, but rather as an impaired or dysregulated immune system in the patient. Close observation and accurate diagnosis of changes in the skin of patients with lung cancer are important when evaluating their immune status and considering their therapy and prognosis.

Disclosure

The authors declare that there are no conflicts of interest.

## References

[1] Habel LA , Ray GT , Silverberg MJ *et al* The epidemiology of herpes zoster in patients with newly diagnosed cancer. Cancer Epidemiol Biomark Prev 2013; 22 (1): 82–90. 10.1158/1055-9965.EPI-12-0815 23118142

[2] Hata A , Kuniyoshi M , Ohkusa Y . Risk of herpes zoster in patients with underlying diseases: A retrospective hospital‐based cohort study. Infection 2011; 39 (6): 537–44. 21800108 10.1007/s15010-011-0162-0 PMC3218277

[3] Choi JY , Kim M , Keam B *et al* The risk of herpes zoster in patients with non‐small cell lung cancer according to chemotherapy regimens: Tyrosine kinase inhibitors versus cytotoxic chemotherapy. Cancer Res Treat 2019; 51 (1): 169–77. 29621875 10.4143/crt.2017.491 PMC6333989

[4] Merselis JG Jr , Kaye D , Hook EW . Disseminated herpes zoster. Arch Intern Med 1964; 113: 679–86. 14120593 10.1001/archinte.1964.00280110059012

[5] McCrary ML , Severson J , Tyring SK . Varicella zoster virus. J Am Acad Dermatol 1999; 41: 1–14. 10411403 10.1016/s0190-9622(99)70398-1

[6] Chairatchneeboon M , Kim EJ . Cutaneous paraneoplastic syndromes In: Kang S, Amagai M, Bruckner AL *et al* (eds). Fitzpatrick's Dermatology, 9th edn McGraw‐Hill Education, New York, NY 2019; 2441–64.

[7] HuSC CGS , Wu CS , Chai CY , Chen WT , Lan CC . Rates of cutaneous metastases from different internal malignancies: Experience from a Taiwanese medical center. J Am Acad Dermatol 2009; 60: 379–87. 19056145 10.1016/j.jaad.2008.10.007

[8] Hidaka T , Ishii Y , Kitamura S . Clinical features of skin metastasis from lung cancer. Intern Med 1996; 35: 459–62. 8835596 10.2169/internalmedicine.35.459

[9] Mahale P , Yanik EL , Engels EA . Herpes zoster and risk of cancer in the elderly U.S. population. Cancer Epidemiol Biomark Prev 2016; 25 (1): 28–35. 10.1158/1055-9965.EPI-15-1033 PMC4713252 26578536

[10] Cho HG , Zehnder JL , Lee YK , Lim H , Kim M . Increased risk of lymphoid malignancy in patients with herpes zoster: A longitudinal follow‐up study using a national cohort. BMC Cancer 2019; 19: 1148. 31775678 10.1186/s12885-019-6349-y PMC6882027

[11] Liu YC , Yang YH , Hsiao HH *et al* Herpes zoster is associated with an increased risk of subsequent lymphoid malignancies ‐a nationwide population‐based matched‐control study in Taiwan. BMC Cancer 2012; 12: 503. 23114019 10.1186/1471-2407-12-503 PMC3531246

[12] Mckay SD , Guo A , Pergam SA , Dooling K . Herpes zoster risk in immunocompromised adults in the United States: A systematic review. Clin Infect Dis 2019; 71 (7): e125–e134. 10.1093/cid/ciz1090 PMC7195255 31677266

[13] Juel‐Jensen BE . The natural history of shingles. Events associated with reactivation of varicella‐zoster virus. J R Coll Gen Pract 1970; 20 (101): 323–7. 5533234 PMC2236212

[14] Lookingbill DP , Spangler N , Sexton FM . Skin involvement as the presenting sign of internal carcinoma. A retrospective study of 7316 cancer patients. J Am Acad Dermatol 1990; 22: 19–26. 2298962 10.1016/0190-9622(90)70002-y

[15] Mollet TW , Gracia CA , Koester G . Skin metastases from lung cancer. Dermatol Online J 2009; 15 (5): 1. 19624979

[16] Schmidt SAJ , Mor A , Schønheyder HC , Sørensen HT , Dekkers OM , Cronin‐Fenton D . Herpes zoster as a marker of occult cancer: A systemic review and meta‐analysis. J Infect 2017; 74 (3): 215–35. 27845154 10.1016/j.jinf.2016.11.005

